# Immunologic and Clinical Failure of Antiretroviral Therapy in People Living with Human Immunodeficiency Virus within Two Years of Treatment

**DOI:** 10.1155/2020/5474103

**Published:** 2020-05-03

**Authors:** Solomon Weldegebreal Asgedom, Mahlet Maru, Beletu Berihun, Kidu Gidey, Yirga Legesse Niriayo, Tesfay Mehari Atey

**Affiliations:** School of Pharmacy, College of Health Sciences, Mekelle University, Ethiopia

## Abstract

**Background:**

Early initiation of highly active antiretroviral therapy (HAART) decreases human immunodeficiency virus- (HIV-) related complications, restores patients' immunity, decreases viral load, and substantially improves quality of life. However, antiretroviral treatment failure considerably impedes the merits of HAART.

**Objective:**

This study is aimed at determining the prevalence of immunologic and clinical antiretroviral treatment failure.

**Methods:**

A cross-sectional study design using clinical and immunologic treatment failure definition was used to conduct the study. Sociodemographic characteristics and clinical features of patients were retrieved from patients' medical registry between the years 2009 and 2015. All patients who fulfilled the inclusion criteria in the study period were studied. Predictors of treatment failure were identified using Kaplan-Meier curves and multivariable Cox regression analysis. Data analysis was done using SPSS version 21 software, and the level of statistical significance was declared at a *p* value < 0.05.

**Results:**

A total of 770 were studied. The prevalence of treatment failure was 4.5%. The AZT-based regimen (AHR = 16.95, 95% CI: 3.02-95.1, *p* = 0.001), baseline CD4 count ≥ 301 (AHR = 0.199, 95% CI: 0.05-0.76, *p* = 0.018), and bedridden during HAART initiation (AHR = 0.131, 95% CI: 0.029-0.596, *p* = 0.009) were the predictors of treatment failure.

**Conclusion:**

The prevalence of treatment failure was lower with the risk being higher among patients on the AZT-based regimen. On the other hand, the risk of treatment failure was lower among patients who started HAART at baseline CD4 count ≥ 301 and patients who were bedridden during HAART initiation. We recommend further prospective, multicenter cohort studies to be conducted to precisely detect the prevalence of treatment failure using viral load determination in the whole country.

## 1. Introduction

Human immunodeficiency virus (HIV)/acquired immune disease is a debilitating disease that resulted in modern global crisis. The disease is not curable, and it has no medication that can cure yet [1]. Globally, there was a total estimate of 36.9 million people living with HIV in 2017 while about 21.7 million people were receiving antiretroviral therapy (ART). In Ethiopia, from 610,000 people who lived with HIV, 437,000 people were receiving ART making ART coverage 71% in 2017 [2]. In developed countries, antiretroviral effectiveness has been successfully demonstrated [3]. However, there are few studies conducted in developing countries with scarce resources declaring effectiveness of ART [4]. ART drastically improves survival and quality of life of HIV patients [5–7], markedly declines AIDS progression, reduces the incidence of infections and hospitalization, and decreases the incidence of organ function complications [8, 9].

Although highly active antiretroviral therapy (HAART) improved patients' health outcome substantially, a significant number of people on the first-line HAART failed to achieve the required virological suppression [10]. As ART coverage increased, simultaneously, it escalated resistance to ART [11]. In Ethiopia, immunologic failure among pediatric patients was found to be 10% [12, 13]. Moreover, a study conducted among adult HIV patients reported prevalence of 22% from one study in the country [14]. In the southern part of the country, an immunologic failure of 17.6% was also detected [15]. Other studies from Bahirdar, Northwest Ethiopia, Debremarkos, Northwest Ethiopia, and Gonder, Northwest Ethiopia, found immunologic and clinical treatment failures in 10.7%, 21%, and 4.1% of the studied patients, respectively [16–18].

Failure to the first-line HAART regimen is perilous, because the second-line HAART regimens are dearth and unaffordable [19–24]. Recent estimates suggest that, although 2% of patients on ART are on a second-line regimen [25], a number of patients are likely to be failed. Despite the failed first-line HAART regimen, many patients have not been switched to the second-line regimen [10]. A proportion of HIV/AIDS patients who need the second-line and third-line therapies continued to grow up with improvement in survival of the patients. However, the second-line and third-line HAART regimens are scarce which currently threaten patients [26].

HIV/AIDS patients faced challenges when a patient had stayed longer on the failed HAART regimen [24]. To overcome the problem, guidelines have been developed by the World Health Organization (WHO) to be implemented in developing countries for patients on follow-up and to monitor immunologic, clinical, and virologic response of patients to their treatment [10]. Despite its importance, monitoring virologic response of ART is a big challenge, specifically in low-income nations. In developing countries like Ethiopia, the detection, timely monitoring, and management of a patient who failed his first-line regimen are compromised and are also considered an immense problem [27, 28]. However, for resource-limited health care settings, the WHO guideline has been developed and distributed parameters for clinical and immunological monitoring of ART response [29]. In Ethiopia, viral load testing is not available, unaffordable, and not pragmatic as a routine practice in many health care settings including the study area; instead, immunological and clinical monitoring is routinely used. Treatment failure and its contributing factors in Ethiopia specifically in the study area are not precisely known. This study, therefore, investigated prevalence of clinical and immunologic failure of people living with HIV after two years of ART use in Northern Ethiopia.

## 2. Methods

### 2.1. Study Area

The study was conducted in the largest hospital of Tigray region found in Mekelle city, which is named Ayder Comprehensive Specialized Hospital (ACSH). Mekelle is the capital city of Tigray region in Northern Ethiopia which is located 783 km away from the capital of Ethiopia, Addis Ababa. ACSH is a comprehensive specialized hospital which serves a higher number of patients in the northern district of the country. The hospital is a teaching as well as a comprehensive specialized hospital. The hospital delivers service for patients referred from Afar, Eritrean refuges and referred patients, and patients from Amhara region in addition to the patients referred from various hospitals of the region. The hospital has a HIV/AIDS clinic, which gives free clinical service, counseling, and care for people living with HIV/AIDS.

### 2.2. Study Design and Study Population

An institutional-based cross-sectional study design was used to conduct the study. People living with HIV who started HAART and patients who were regularly followed at the HIV clinic of ACSH were the source population in our study. Patients who were registered at the ACSH HIV clinic and on follow-up from 2009 to 2015 that fulfilled the inclusion criteria were the study population. We included all HIV patients who had regular follow-up at ACSH in the years between 2009 and 2015. On the other way, we excluded HIV patients who were younger than 16 years, transferred outpatients, transferred inpatients, HIV patients with incomplete data registry, patients who were lost, patients who dropped from follow-up, and HIV patients who had started HAART on 2016. HIV patients were followed starting from the day of naïve HAART initiation until the time of treatment failure or censor retrospectively. Survival time was measured in days from the date of initiation of HAART until the time of treatment failure/censor. HIV patients who had evidence of treatment failure were declared to be failures and patients who did not have treatment failure were considered censored. A total of 770 out of 2134 patient registries of HIV patients who started HAART from 2009 up to 2015 were included and studied. The duration of the patients' follow-up period was 2 consecutive years starting from the year of HAART initiation.

Clinical and/or immunologic treatment failure was the primary outcome of the study and was our dependent variable. The WHO clinical and immunologic failure definition was used by physicians in the HIV clinic to declare the treatment failure. Based on the WHO guideline, treatment failure was defined as the fall of CD4 count to baseline (or below), CD4 levels persisting below 100 cells/mm, 50% fall from the treatment peak value, or development of new opportunistic infections [30]. Sex, place of residence, age, baseline weight, baseline CD4 count, baseline TB symptoms, baseline WHO stage, eligibility criteria, year of HAART initiation, baseline functional status, and ART regimen were the independent variables studied.

### 2.3. Data Collection Tool and Procedure

The Ethical Review Board, College of Health Sciences, Mekelle University, offered us an ethical clearance and approval of the study. Informed written patient consent was obtained from patients prior to data collection. Moreover, to obtain patients' data, permission was granted from the ACSH medical director and HIV/AIDS clinic. We also confirm that all methods were performed in accordance with the relevant guidelines and regulations. The HIV/AIDS patients' medical registry review was entirely confidential. The standardized HIV entry and follow-up form employed by the HIV clinic was adapted and was used to extract data from patients' medical registries. All the data was collected from the patients' medical registries, follow-up form, and patients' medical card. Two pharmacists who were working outside the hospital were recruited, trained for two days, and collected patients' data. Prior to data collection, a pretest was conducted on 39 patients. Patients who were involved in the pretest assessment were excluded from data analysis. Based on the pretest finding, amendments were done on the data collection format. Close supervision and follow-up on the data collection process were made by the principal investigator.

### 2.4. Statistical Analysis

SPSS version 21 software was used for data analysis. Data was refined and checked for consistency and completeness. After the data was profoundly assessed, the data was analyzed. Before data synthesis and analysis, the data was entered and cleaned via the principal investigator and one additional data clerk. Survival analysis was deployed using the Kaplan-Meier model. The level of significance of survival curves was compared through Log-Rank (Mantel-Cox), Breslow (Generalized Wilcoxon), and Tarone-W. Factors associated with treatment failure, and its predictors were identified using Cox regression proportional hazard model analysis. Meanwhile, the level of significance was deemed to be significant at *p* value less than 0.05.

## 3. Results

### 3.1. Demographic and Clinical Characteristics of Patients

A total of 770 people who were living with HIV were analyzed. Majority (442 (57.4%)) of the patients were females. The average age of patients during initiation of ART was 32.9 ± 9.5 [mean ± SD] years. On ART initiation, 410 (53.2%) patients were WHO stage three (T3). Besides, TDF+3TC+EFV was the most frequently prescribed HAART regimen which was prescribed for 312 (40.5%) patients (Table 1).

### 3.2. Treatment Failure and Its Predictors

**Figure 1:**
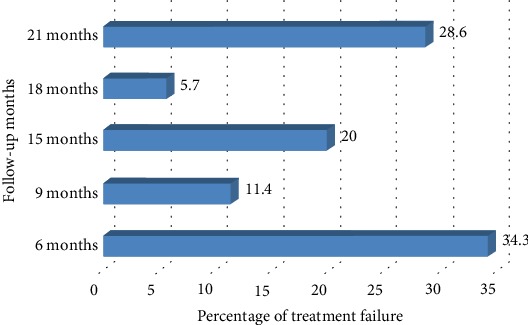
Percentage of treatment failure among people living with HIV in ACSH, Northern Ethiopia, 2017.

The median time to ART failure was 15 months, whereas the mean (±SD) time to fail the treatment was 13.11 ± 6.35 months. The prevalence of treatment failure was calculated to be 4.5%. From the 35 patients who failed to treatment, 14 patients had started TDF+3TC+EFV/NVP, thirteen patients started AZT+3TC+NVP/EFV, and 8 patients had started D4T+3TC+NVP/EFV regimens. Majority (34.3% (12)) of the patients' treatment failure occurred within 6 months, and 28.6% (10) of ART failure were detected after 21 months of consecutive therapy. No record of treatment failure was found within the 12^th^ and 24^th^ months of follow-up (Figure 1).

**Figure 2:**
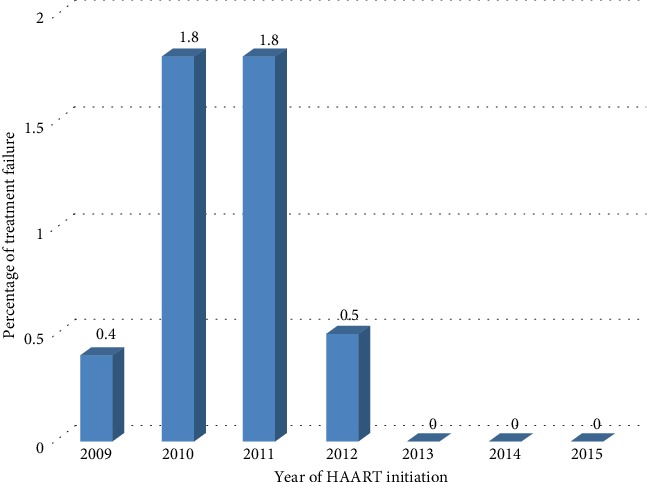
Distribution of treatment failure via the year of HAART initiation among people living with HIV in ACSH, Northern Ethiopia, 2017.

All (100%) antiretroviral treatment failures were identified in the years 2009-2013. In 2014 and 2015, no evidence of treatment failure was detected. Majority (1.8%) of the treatment failures occurred in the years between 2010 and 2011, 0.5% in 2012, and 0.4% in 2009 (Figure 2).

The zidovudine-based regimen (Log-Rank (Mantel-Cox) (*p* < 0.001)), baseline CD4 count (Log-Rank (Mantel-Cox) (*p* = 0.012)), sex (Log-Rank (Mantel-Cox) (*p* = 0.004)), baseline functional status (Log-Rank (Mantel-Cox) (*p* = 0.013)), baseline WHO stage (Log-Rank (Mantel-Cox) (*p* = 0.004)), and baseline TB symptoms (Log-Rank (Mantel-Cox) (*p* = 0.001)) were variables that were significantly associated with treatment failure.

The AZT-based regimen, baseline CD4 count, and baseline functional status were the factors that were found to be predictors of treatment failure on multivariate Cox regression analysis. Patients who started the zidovudine-based regimen (AHR = 16.95, 95% CI: 3.02-95.1, *p* = 0.001) were 17 times more likely to have treatment failure than patients who started the nonzidovudine-based ART regimen. Patients with baseline CD4 count ≥ 301 cells/mm^3^ (AHR = 0.199, 95% CI: 0.05-0.76, *p* = 0.018) were 80% less likely to fail their treatment than patients who had baseline CD4 count ≤ 200 cells/mm^3^. Moreover, patients who were bedridden at baseline (AHR = 0.131, 95% CI: 0.029-0.596, *p* = 0.009) were 87% less likely to fail their therapy than patients who were ambulatory during HAART initiation (Table 2).

## 4. Discussion

Despite the significant change in the era of HIV management from killer to a chronically manageable disease, another problem, which is ART failure, began and continued to be the hurdle for substantial control of the HIV/AIDS disease. The extant of resistance HIV strains to the first-line ART regimens leads patients to more expensive regimens and to use medications that have less tolerable toxicities and are less effective [20]. Treatment failure in combination with other concomitant problems had remained the main challenge for successful HIV treatment and milestone achievement globally. It is vital to determine the prevalence of treatment failure, identify factors that boost ART failure, and find out strategies that could improve the problem quickly. In this study, we mainly aimed to determine prevalence of treatment failure in the specified setup. According to our calculation, 4.5% prevalence of antiretroviral treatment failure was discerned over the entire the 24-month follow-up period. With regard to the factors of ART failure, patients who were on the AZT-based regimen, baseline CD4 count, and baseline functional status were the three predictors of treatment failure.

Our study found 4.5% prevalence of treatment failure among the studied patients. This finding is quite lower when compared to treatment failures reported in Gonder, Ethiopia (11.5%) [14], Southwest Ethiopia (20.3%) [11], Northern Ethiopia (22%) [13], Addis Ababa (8.2%) [11], and Western Ethiopia (11.5%) [12]. The discrepancy could be due to the differences of study population and follow-up periods. The studies in Addis Ababa [11] and Jimma [12] were done among children or people living with HIV. The studies also had significant variation in methods of ART failure detection used and length of follow-up periods [11–13]. Moreover, in some sub-Saharan African countries, an overall higher immunological failure that ranged from 10% to 32% was reported in four studies [31, 32]. Furthermore, one study from Nigeria reported an immunologic antiretroviral treatment failure of 32% [33]. In addition to heterogeneity in methodology, low detection skill of health care professionals to identify treatment failure and lower patients' registry documentation might contribute for lesser detection of treatment failure in our study. In developing countries, many patients died at their home and the patients who died at home might not be documented in the patients' chart appropriately.

Patients who started the AZT-based regimen were 17 times more likely to develop treatment failure than patients who started with the non-AZT-based regimen. This finding concurred with other studies from Ethiopia. One study from Ethiopia found higher risk of mortality with the AZT-based HAART regimen as compared to the TDF-based regimen [34]. Case-control studies done in Kenya also depicted that ART clients who were taking the zidovudine-based regimen experienced more treatment failures as compared to other regimen-based treatments [35, 36]. In terms of virological load suppression, both regimens had similar viral load suppression [34]. On the contrary, immunological response which was demonstrated in terms of the rise in CD4 count was lowest in the AZT-based regimen as compared to the TDF-based regimen [34]. Thus, the lower potential of the CD4 count scale-up of the AZT-based regimen might lead patients to develop opportunistic infections, which ultimately insist declaration of immunologic antiretroviral treatment failure [37]. The AZT-based regimen was the first-line regimen before the TDF-based regimen was approved as the first line. Besides utilization of AZT at higher levels in the first 6 months of initiation, anemia and rash were the most common reason accounting for treatment change in many patients [38]. Therefore, drug toxicity might lead patients to be nonadherent to their medication which could lead them to treatment failure.

Patients who had baseline CD4 count ≥ 301cells/mm^3^ (AHR = 0.199, 95% CI: 0.05-0.76, *p* = 0.018) were 80% less likely to fail their treatment than patients who had baseline CD4 count ≤ 200 cells/mm^3^. There are studies that reported that lower CD4 count has been associated with higher odds of treatment failure [11, 39]. In low-income nations, use of CD4 count was used for treatment decisions, for regimen switch, and as a predictor of disease progression. It was highly used as a criterion for HAART initiation and as a marker of treatment outcomes [40]. CD4 count change after the initiation of ART is known to be a good predictor of Health-Related Quality of Life (HRQL) [41, 42]. CD4 count also reduces risk of hospitalization prominently [38]. Thus, patients who started at elevated CD4 count are less likely to develop opportunistic infections, hence less likely to develop clinical and immunologic failures.

Patients who were bedridden at the time of HAART initiation (AHR = 0.131, 95% CI: 0.029-0.596, *p* = 0.009) were 87% less likely to fail their treatment regimen than patients who were ambulatory. Similar to this finding, studies from the USA reported that advanced clinical stages predicted improvement in CD4 count change. The lower risk of treatment failure among bedridden patients could be due to better care and ART adherence of the patients. When a patient has poor adherence to antiretroviral therapy and cotrimoxazole prophylaxis therapy, there will be a significant reduction in CD4 count by 111.2 and 60.88 times, respectively, than good adherence to ART and cotrimoxazole prophylaxis therapy [42, 43]. Ergo, when a patient is adherent to his medication, it will likely increase the CD4 count which would hinder treatment failure on the other side [11].

Our study is not without flaw; hence, it has some limitations. We used the patients' registry as our main source of data. Therefore, both the ART database and ART patients' chart are the secondary sources; thus, all the problems related to the use of the secondary data apply to this study. Moreover, some important predictors like CPT adherence and AST and ALT levels due to poor patient medical card documentation were not collected. Despite some limitations, this study included a representative number of patients to elucidate the issue.

## 5. Conclusion

The prevalence of treatment failure was significantly lower when compared to many local and global studies. High risk of treatment failure was found among patients on the AZT-based regimen. Low risk of treatment failure was found among patients who started HAART at baseline CD4 count ≥ 301. Moreover, patients who were bedridden during initiation of HAART were less likely to develop treatment failure. To precisely detect the prevalence of treatment failure which could be generalized to general population, a prospective cohort multicenter study is recommended.

## Figures and Tables

**Table 1:** Demographic and clinical characteristics of HIV patients in Ayder Comprehensive Specialized Hospital, 2017 (*n* = 770).

Variable		Frequency (%)
Age (in year)	16-29	294 (38.2)
	30-44	390 (50.6)
	≥44	86 (11.2)

BMI (in kg/m^2^)	<18	286 (37.1)
	18-24.9	429 (55.7)
	25-34	55 (7.1)

Sex	Female	442 (57.4)
	Male	328 (42.6)

Residence	Rural	190 (24.7)
	Urban	580 (75.3)

Why eligible	Clinical only	45 (5.8)
	CD4 count	547 (71.0)
	Others	178 (23.2)

Baseline functional status	Ambulatory	203 (26.4)
	Working	487 (63.2)
	Bedridden	80 (10.4)

Baseline WHO stage	T1	113 (14.7)
	T2	97 (12.6)
	T3	410 (53.2)
	T4	150 (19.5)

Baseline TB screen	Positive	167 (21.7)
	Negative	603 (78.3)

Baseline ARV regimen	TDF+3TC+EFV	312 (40.5)
	AZT+3TC+NVP	235 (30.5)
	D4T+3TC+NVP	100 (13.0)
	TDF+3TC+NVP	79 (10.3)
	D4T+3TC+EFV	26 (3.4)
	AZT+3TC+EFV	18 (2.3)

Others: TB, HBV, and pregnancy.

**Table 2:** Predictors of treatment failure among people living with HIV in ACSH, Northern Ethiopia, 2017.

Variables		Treatment failure		AHR	95% CI	*p* value
		No (%)	Yes (%)			
ART regimen	Non-AZT-based	495 (67.3)	22 (62.9)	1	1	1
	AZT-based	240 (32.7)	13 (371)	16.95	3.02-95.1	0.001^∗^

Baseline CD4 count	≤200	539 (73.3)	26 (74.3)	1	1	1
	200-300	124 (16.9)	3 (8.6)	1.25	0.26-6.02	0.781
	≥301	72 (9.8)	6 (17.1)	0.199	0.05-0.76	0.018^∗^

Sex	Female	422 (57.4)	20 (57.1)	1	1	1
	Male	313 (42.6)	15 (42.9)	0.622	0.27-1.45	0.272

Baseline functional status	Ambulatory	185 (25.2)	18 (51.4)	1	1	1
	Working	474 (64.5)	13 (37.1)	1.04	0.29-3.47	0.995
	Bedridden	76 (10.3)	4 (11.4)	0.131	0.029-0.596	0.009^∗^

Baseline WHO stage	T1 and T2	208 (28.3)	2 (5.7)	1	1	1
	T3	391 (53.2)	19 (54.3)	0.915	0.155-5.4	0.922
	T4	136 (18.5)	14 (40)	3.4	0.42-27.52	0.251

Note: AHR: adjusted hazard ratio; CI: confidence interval; ART: antiretroviral therapy; WHO: World Health Organization; AZT: zidovudine. ^∗^Statistical significance at *p* < 0.05.

## Data Availability

The datasets supporting the conclusions of the study are included in the article. Any additional data will be available on request.
